# Clinicopathological characteristics and cancer-specific prognosis of primary pulmonary lymphoepithelioma-like carcinoma: a population study of the US SEER database and a Chinese hospital

**DOI:** 10.3389/fonc.2023.1103169

**Published:** 2023-05-19

**Authors:** Qun Zhang, Yuan Dai, Linling Jin, Shuangshuang Shi, Chang Liu, Rong Rong, Wenkui Sun, Shanlin Dai, Hui Kong, Weiping Xie

**Affiliations:** ^1^ Department of Respiratory and Critical Care Medicine, The First Affiliated Hospital of Nanjing Medical University, Nanjing, Jiangsu, China; ^2^ Department of Pathology, The First Affiliated Hospital of Nanjing Medical University, Nanjing, China

**Keywords:** primary pulmonary lymphoepithelioma-like carcinoma (PPLELC), cancer-specific survival, incidence, SEER, NSCLC

## Abstract

**Introduction:**

Primary pulmonary lymphoepithelioma-like carcinoma (PPLELC) is a rare histological type of non-small cell lung cancer (NSCLC), which accounts for less than 1% of NSCLC. Currently, there is no well-recognized treatment guideline for PPLELC.

**Methods:**

We identified PPLELC patients from the Surveillance, Epidemiology, and End Results (SEER) dataset between 2000 and 2015 (n = 72) as well as from our medical center between 2014 and 2020 (n = 16). All diagnoses were confirmed by pathological testing, and the clinicopathological characteristics of patients were retrieved and summarized. Survival analyses were conducted using the Kaplan–Meier analysis and log-rank tests. Multivariate survival analysis was performed with the Cox regression hazards model.

**Results:**

The median age at diagnosis of the PPLELC cohort was 64 years, ranging from 15 to 86 years. The percentages of patients with TNM stages I, II, III, and IV were 52.3%, 10.2%, 20.5%, and 17.0%, respectively. Among the 88 cases, lesion resection was performed in 69 cases (78.4%), 16 cases (18.1%) received beam radiation, and 40 cases (45.5%) underwent chemotherapy. In the SEER dataset of lung cancer, the percentage of PPLELC in the Asian race (0.528‰) was almost 10 times higher than that in the white (0.065‰) and black (0.056‰) races. Patients with TNM stage III–IV exhibited a worse prognosis than those with TNM stage I–II (*p* = 0.008), with a 5-year cancer-specific survival (CSS) rate of 81.8% for TNM stage I–II and 56.2% for TNM stage III–IV. Specifically, the N stage and M stage were the leading prognostic factors, not the T stage and tumor size. Moreover, patients who underwent surgery had significantly better outcomes than those who did not (*p* = 0.014). Additional multivariate analysis indicated that the TNM stage was an independent prognosis factor for CSS (HR, 3.31; 95% CI, 1.08–10.14).

**Conclusion:**

PPLELC is a rare tumor with Asian susceptibility. Although the prognosis of PPLELC is better than that of other subtypes of NSCLC, it remains unsatisfactory for advanced-stage disease. The current treatment options for PPLELC include surgical resection, chemotherapy, radiotherapy, and immune therapy. Among these options, patients with surgical resection have better survival rates in this study. However, large-scale clinical research trials will be necessary to develop effective treatment guidelines for PPLELC.

## Introduction

Lung cancer accounts for the leading cancer-related death worldwide. The disease can be broadly categorized into non-small-cell lung cancer (NSCLC) and small-cell lung cancer (SCLC). Though subtype-specific morbidity and mortality had been extensively reported, some rare pathological types of lung cancer, such as primary pulmonary lymphoepithelioma-like carcinoma (PPLELC), have not been fully characterized in terms of incidence and prognosis (1). PPLELC is a rare histological subtype of NSCLC, and further research is needed to understand its clinical features.

PPLELC was first reported by Begin et al. in 1987 on a 40-year-old woman of Southeast Asian descent in Canada (2). This epithelial tumor, which is associated with Epstein–Barr (EB) virus infection, is histologically similar to nasopharyngeal carcinoma (NPC) (3). In 2004, the World Health Organization (WHO) initially classified PPLELC as a subtype of large cell carcinoma, but in the 2005 classification, it was re-categorized as “other and unclassified carcinomas” of NSCLC (4). Studies have indicated that PPLELC was typically diagnosed at an early stage and has a better prognosis than other NSCLC subtypes (5, 6). However, the majority of reported PPLELC cases were from Asia, and limited studies have focused on its incidence and prognosis in Western countries (7, 8).

Over the past decade, our center has treated numerous cases of PPLELC. Our study aimed to examine the clinicopathological characteristics, treatment methods, and cancer-specific prognosis of PPLELC in both the United States and our own medical center.

## Patients and methods

### Patient selection

We identified lung cancer patients in Surveillance, Epidemiology, and End Results (SEER) dataset between 2000 and 2015 who had been diagnosed with ICD-O-3 code as “8082/3: lymphoepithelial carcinoma”. All cases were confirmed as primary tumors with positive histology and no previous history of malignancy.

Another additional retrospective cohort of 18 cases was collected from Jiangsu People’s Hospital (JSPH, Nanjing, China) during 2014–2020. All diagnoses in this cohort were based on pathological testing. Two cases were excluded from this cohort due to a history of gastric cancer at the time of their PPLELC diagnoses.

### DNA sequencing

The DNA extraction kit (Kai Shuo) was used to extract genomic DNA (gDNA) from the tissue, following the manufacturer’s instructions. Library construction was carried out using a probe hybridization capture method, with commercial reagents and customized probes. Fragmentation enzymes were employed to shear 15–200 ng of gDNA into 200–350 bp. The constructed DNA libraries were loaded on the NovaSeq 6000 platform (Illumina, San Diego, CA, USA), and the sequences were generated as 150-bp paired-end reads. The resulting base calls were converted to FASTQ files. Adapter trimming and filtering of low-quality bases were performed using the fastp tool (v.2.20.0), and duplicate reads from PCR were eliminated using Dedup with Error Correct. Variants were filtered against common single-nucleotide polymorphisms (SNPs) in public databases, while copy number variations (CNVs) and fusions were conducted with CNVkit (dx1.1) and factera (v1.4.4), respectively (9, 10).

### Statistics

SPSS Version 24.0 software was used for data analysis. The Kaplan–Meier method and log-rank test were used for univariate survival analyses. The Cox regression hazards model was used for multivariate survival analysis. SPSS Version 23.0 software was applied for statistical analysis. *p* < 0.05 was considered statistically significant.

### Ethics

This study was approved by the Institutional Review Board of The Ethic Committee of Nanjing Medical University First Affiliated Hospital.

## Results

### Incidence of PPLELC in the SEER dataset

**Figure 1:**
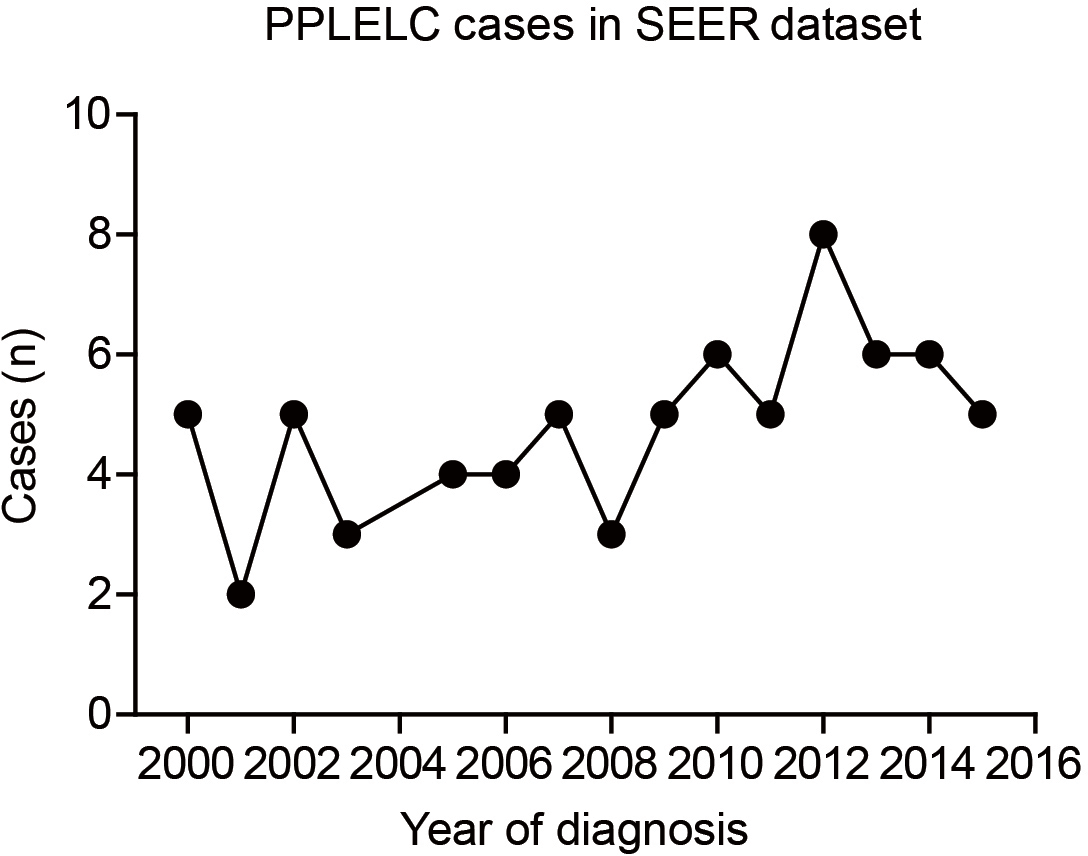
Case numbers of PPLELC in SEER dataset according to diagnostic year. PPLELC, primary pulmonary lymphoepithelioma-like carcinoma; SEER, Surveillance, Epidemiology, and End Results.

As shown in **Figure 1**, there were only two to eight cases diagnosed as PPLELC in the SEER dataset every year, accounting for less than 0.01% of all lung cancers. This highlighted the rarity of the PPLELC subtype. According to the SEER dataset, there were 816,436 cases of diagnosed lung cancer during 2000–2015. Among them, 0.065‰ (44/679,436) were white patients diagnosed with PPLELC, 0.056‰ (5/88,611) were black patients, and 0.528‰ (23/43,571) cases were Asian–Pacific Islanders (APIs) (**Table 1**). This indicated a higher proportion of the PPLELC subtype in lung cancer among APIs.

**Table 1:** PPLELC proportion in lung cancer of different races in SEER dataset.

Races	PPLELC	Lung cancer (LC)	Percentage (PPLELC/LC)
White	44	679,436	0.065‰
Black	5	88,611	0.056‰
Asian–Pacific Islander	23	43,571	0.528‰

PPLELC, primary pulmonary lymphoepithelioma-like carcinoma; SEER, Surveillance, Epidemiology, and End Results.

### Clinical and demographic characteristics of PPLELC cases

A total of 72 patients in the SEER dataset and 16 cases in the JSPH cohort were included in our study. All cases were diagnosed with pathological tests, and their basic characteristics are listed in **Table 2**. The median age of diagnosis for the two PPLELC cohorts was 64 years, ranging from 15 to 86 years. The incidence was similar between women (48.9%) and men (51.1%). More cases exhibited right lung laterality (47/88, 53.4%) than left lung (39/88, 44.3%). All 88 cases were pathologically classified as poorly differentiated or undifferentiated, except for two that were moderately differentiated. More than half of the cases were diagnosed as TX/T1 stage (55.7%), while the percentages were 26.1%, 10.2%, and 8.0% for the T2, T3, and T4 stages, respectively. Most cases (54/88, 61.4%) exhibited negative lymph node metastasis, while the other 34 cases (38.6%) showed positive lymph node metastases. Among the 34 cases above, 11 cases were classified as N1 stage, 21 cases as N2 stage, and 2 cases as N3 stage. The distant metastasis rate was 17.0% in the PPLELC cohort, as reflected by the M1 stage. Accordingly, the percentages of patients with TNM stage I, II, III, and IV were 55.7%, 10.2%, 20.5%, and 17.0%, respectively. As for the disease treatment, we retrieved the therapeutic information including surgery resection, radiotherapy, and chemotherapy. Among the 88 cases, 69 cases (78.4%) underwent lesion resection, 16 cases (18.1%) received beam radiation, and 40 cases (45.5%) accepted chemotherapy.

**Table 2:** Clinical and demographic characteristics of PPLELC cases.

Variables	Total no. (%)	JSPH no. (%)	SEER no. (%)
Total	88 (100%)	16 (100%)	72 (100%)
Age (years)			
Median (range)	64 (15–86) years	55 (39–74) years	66 (15–86) years
≤65 years	49 (55.7%)	14 (87.5%)	35 (48.6%)
>65 years	39 (44.3%)	2 (12.5%)	37 (51.4%)
Sex			
Female	43 (48.9%)	8 (50.0%)	35 (48.6%)
Male	45 (51.1%)	8 (50.0%)	37 (51.4%)
Race			
White	44 (50.0%)	0 (0%)	44 (61.1%)
Black	5 (5.7%)	0 (0%)	5 (6.9%)
Asian–Pacific Islander	39 (44.3%)	16 (100%)	23 (31.9%)
Primary site			
Upper lobe	40 (45.5%)	3 (18.8%)	37 (51.4%)
Lower lobe	29 (33.0%)	6 (37.5%)	23 (31.9%)
Others^#^	19 (21.6%)	7 (43.8%)	12 (16.7%)
Laterality			
Left lung	39 (44.3%)	7 (43.8%)	32 (44.4%)
Right lung	47 (53.4%)	9 (56.3%)	38 (52.8%)
Paired laterality	2 (2.3%)	0 (0%)	2 (2.8%)
Tumor size (cm)			
Median (range)	2.4 (0.5–9.5)	2.2 (0.8–5.6)	2.5 (0.5–9.5)
≤2.5 cm	38 (43.2%)	10 (62.5%)	28 (38.9%)
>2.5 cm	28 (31.8%)	4 (25.0%)	24 (33.3%)
Unknown	22 (25.0%)	2 (12.5%)	20 (27.8%)
T stage			
TX/T1	49 (55.7%)	12 (75.0%)	37 (51.4%)
T2	23 (26.1%)	1 (6.3%)	22 (30.6%)
T3	9 (10.2%)	2 (12.5%)	7 (9.7%)
T4	7 (8.0%)	1 (6.3%)	6 (8.3%)
N stage			
N0	54 (61.4%)	11 (68.8%)	43 (59.7%)
N1	11 (12.5%)	1 (6.3%)	10 (13.9%)
N2	21 (23.9%)	4 (25.0%)	17 (23.6%)
N3	2 (2.3%)	0 (0%)	2 (2.8%)
M stage			
M0	73 (83.0%)	14 (87.5%)	59 (81.9%)
M1	15 (17.0%)	2 (12.5%)	13 (18.1%)
TNM stage^*^			
I	46 (55.7%)	11 (68.8%)	35 (48.6%)
II	9 (10.2%)	0 (0%)	9 (12.5%)
III	18 (20.5%)	3 (18.8%)	15 (20.8%)
IV	15 (17.0%)	2 (12.5%)	13 (18.1%)
Surgery resection			
Yes	69 (78.4%)	14 (87.5%)	54 (75.0%)
No/unknown	20 (22.7%)	2 (12.5%)	18 (25.0%)
Radiotherapy			
Beam radiation	16 (18.1%)	1 (6.3%)	15 (20.8%)
No/unknown	72 (81.8%)	15 (93.8%)	57 (79.2%)
Chemotherapy			
Yes	40 (45.5%)	10 (62.5%)	30 (41.7%)
No/unknown	48 (54.5%)	6 (37.5%)	42 (58.3%)

PPLELC, primary pulmonary lymphoepithelioma-like carcinoma; SEER, Surveillance, Epidemiology, and End Results; JSPH, Jiangsu People’s Hospital.

^#^ Included 2 main bronchus localization, 5 middle lobe localization, 1 overlapping localization, and 3 unspecified localization.

^*^ p < 0.05 by log-rank test.

Among the JSPH cohort, only two cases (2/16, 12.5%) were diagnosed at the age of 65 or older, which was significantly different compared with the SEER dataset (37/72, 51.4%). Interestingly, 43.8% (7/16) of cases showed tumor location within the middle lobe of the right lung. In contrast, only 6.9% (5/72) of SEER cases showed this tumor location, and all of them were APIs. This difference seems to be related to race since none of the white or black patients exhibited right middle lobe lung tumor location. However, further validation will be necessary due to the limited number of cases in this study. Another difference was the smaller tumor size in our cohort. In the JSPH cohort, 62.5% (10/16) of cases were diagnosed with tumor size less than or equal to 2.5 cm in diameter, while it was only 38.9% (28/72) in the SEER cohort. Considering the different diagnostic eras in the SEER cohort (2000–2015) and JSPH cohort (2014–2020), this may be caused by more sensitive CT scans in the recent decade rather than racial differences. Although the percentages of M1 stage cases were comparable in the SEER cohort (13/72, 18.1%) and JSPH cohort (2/16, 12.5%), the treatment selection seemed different. There were more cases that underwent surgery resection (87.5% *vs.* 75.0%) and fewer cases accepted radiotherapy (6.3% *vs.* 20.8%) in the JSPH cohort (**Table 2**).

### Survival analysis of CSS in PPLELC cases

**Figure 2:**
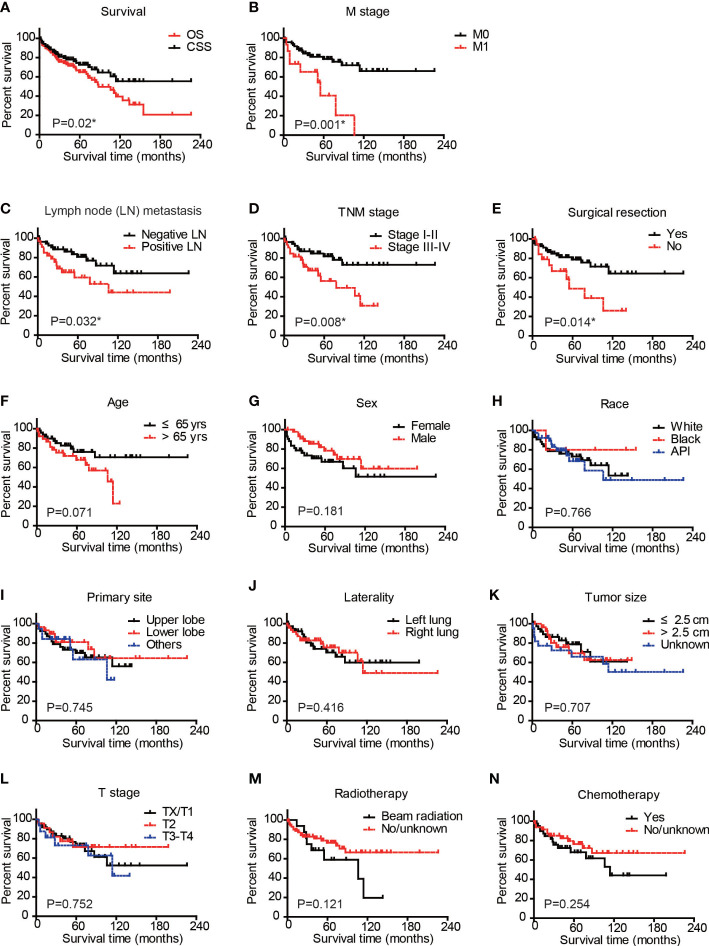
Cancer-specific survival analysis of PPLELC patients. Cancer-specific survival (CSS) and overall survival (OS) of entire cohort **(A)** CSS of patients based on M stage **(B)**, Lymph node metastasis **(C)**, TNM stage **(D)**, surgical resection **(E)**, age **(F)**, sex **(G)**, race **(H)**, primary tumor site **(I)**, tumor laterality **(J)**, tumor size **(K)**, T stage **(L)**, radiotherapy **(M)**, or chemotherapy **(N)**. Data were analyzed by Kaplan–Meier method and log-rank test. * p < 0.05. PPLELC, primary pulmonary lymphoepithelioma-like carcinoma.

The median follow-up time was 51 months, ranging from 1 to 226 months. By the end of the follow-up period, there were 24 cases (24/88, 27.3%) dead due to PPLELC (**Supplementary Table 1**). The 1-, 3-, and 5-year cancer-specific survival (CSS) rates were 90.8%, 79.5%, and 72.6%, respectively (**Figure 2A**). The mean CSS time was 148.7 ± 12.6 months. **Figure 2A** shows a significant difference between the OS and CSS of PPLELC (*p* = 0.02).

We next conducted Kaplan–Meier survival analyses by dividing patients into different subgroups according to clinical and demographic characteristics (**Figures 2B–N**). The M stage of patients significantly affected the CSS of PPLELC (*p* = 0.001, **Figure 2B**). The 5-year CSS rate of patients with M0 stage was 78.5%, while it decreased to 40.7% for patients with M1 stage. The mean CSS time of patients with M0 stage was 166.5 ± 12.8 months, while it decreased to 54.4 ± 11.4 months for patients with M1 stage (**Table 3**). Additionally, the N stage of patients also affected prognosis with a statistically significant difference (*p* = 0.032, **Figure 2C**). The 5-year CSS rate of patients with negative lymph nodes was 80.7%, while it decreased to 59.4% for patients with positive lymph node metastases (**Table 3**). Consistently, patients with TNM stage III–IV exhibited worse prognoses than those in TNM stage I–II (*p* = 0.008, **Figure 2D**). The 5-year CSS rate of patients in TNM stage I–II was 81.8%, while it decreased to 56.2% for patients in TNM stage III–IV. The mean CSS time of cases in TNM stage I–II was 176.3 ± 13.5 months, while it decreased to 80.2 ± 10.5 months for patients in TNM stage III–IV (**Table 3**). Patients who underwent surgical resection showed a 5-year CSS rate of 78.8%, while it was only 48.7% in those without surgical intervention (*p* = 0.0014, **Figure 2E**). The mean CSS time of patients who underwent surgical resection was 164.1 ± 13.9 months, while it decreased to 72.6 ± 13.2 months for patients without surgical treatment (**Table 3**). In contrast, other factors seemed to have no significant effect on PPLELC survival (**Figures 2F–N**, all *p* > 0.05).

**Table 3:** Cancer-specific survival (CSS) of PPLELC cases.

Variables	Case (n)	5-year CSS (%)	Mean CSS (months)	p-Value^a^	HR	95% CI	p-Value^b^
Age (years)				0.071			0.070
≤65 years	49	76.0	170.83 ± 14.6		Reference		
>65 years	39	67.9	81.77 ± 8.0		2.13	0.94–4.83	
Sex				0.181			0.154
Female	43	66.8	137.4 ± 18.3		Reference		
Male	45	78.2	143.5 ± 13.9		0.54	0.23–1.27	
Race				0.766			
White	44	73.0	101.0 ± 9.0		–		
Black	5	80.0	128.0 ± 24.2				
Asian–Pacific Islander	39	68.4	138.6 ± 21.2				
Primary site				0.745			
Upper lobe	40	69.9	101.0 ± 9.4		–		
Lower lobe	29	81.0	163.0 ± 20.3				
Others	19	63.2	83.9 ± 11.0				
Laterality^#^				0.416			
Left lung	39	70.2	135.3 ± 14.6		–		
Right lung	47	75.5	144.1 ± 19.2				
Tumor size (cm)				0.707			
≤2.5 cm	38	78.8	106.5 ± 10.1		–		
>2.5 cm	28	69.4	108.0 ± 11.8				
Unknown	22	65.9	136.9 ± 22.2				
T stage				0.752			
TX/T1	49	72.8	144.6 ± 17.9		–		
T2	23	71.5	149.2 ± 17.0				
T3–T4	16	73.1	94.2 ± 14.4				
N stage				0.032*			
N0	54	80.7	165.9 ± 15.3		–		
N1–N3	34	59.4	110.7 ± 17.0				
M stage				0.001**			
M0	73	78.5	166.5 ± 12.8		–		
M1	15	40.7	54.4 ± 11.4				
TNM stage				0.008**			0.036*
I–II	55	81.8	176.3 ± 13.5		Reference		
III–IV	33	56.2	80.2 ± 10.5		3.31	1.08–10.14	
Surgery resection				0.014*			0.414
Yes	68	78.8	164.1 ± 13.9		Reference		
No/unknown	20	48.7	72.6 ± 13.2		1.412	0.44–4.59	
Radiotherapy				0.121			0.987
Beam radiation	16	58.9	89.0 ± 13.0		Reference		
No/unknown	72	76.4	154.1 ± 13.8		0.99	0.33–3.02	
Chemotherapy				0.254			0.566
Yes	40	67.9	119.1 ± 15.8		Reference		
No/unknown	48	76.4	165.4 ± 15.0		1.71	0.47–6.16	

PPLELC, primary pulmonary lymphoepithelioma-like carcinoma.

^#^ Two cases were recorded with paired laterality and were not shown here due to limited case numbers.

a Data significance was compared by log-rank test.

b Data significance was compared by Cox regression test.

* p < 0.05, ** p < 0.01.

Multivariate analysis was performed using a Cox regression hazards model. The variables with *p* < 0.3 from univariate analyses, including patients’ age, sex, TNM stage, surgery resection, radiotherapy, and chemotherapy (**Table 3**), were subjected to this model. We excluded N and M stages during multivariate analysis due to their inseparable correlation with the TNM stage. As a result, the TNM stage was the only factor that had an independent prognostic effect on PPLELC survival (*p* = 0.036, HR, 3.31; 95% CI, 1.08–10.14). The hazard ratio (95% confidence interval) for age > 65, sex male, no surgery treatment, no radiotherapy, and no chemotherapy was 2.13 (0.94–4.83), 0.54 (0.23–1.27), 1.41 (0.44–4.59), 0.99 (0.33–3.02), and 1.71 (0.47–6.16), respectively.

## Discussion

PPLELC is a rare subtype of unclassified NSCLC, and the presence of the EB virus in tumor cells is critical for disease diagnosis. The clinical characteristics, prognostic factors, and therapies of PPLELC remain unclear. The major reports on PPLELC were primarily from Eastern countries, such as Southern China and Taiwan (8). Our study of the US SEER dataset also showed a high proportion of the PPLELC subtype among Asians with lung cancer, indicating a prevalence of PPLELC among East Asians. It is similar to another EB-associated disease, NPC, which occurs frequently in Southeast Asian countries (11).

Previous research has reported that PPLELC showed a better prognosis than other common subtypes of NSCLC (7). For instance, Jiang et al. reported a 5-year disease-free survival (DFS) rate of 47% from their medical center (12). Moreover, He et al. indicated that the median overall survival time was 107 months using the SEER dataset of 62 PPLELC cases during 1973–2011, and univariate analysis demonstrated that only age > 65 was associated with poor prognosis (13). However, non-lung cancer-related mortality hazard contributed significantly to the overall survival of long-term cancer survivors, such as PPLELC patients (14). **Supplementary Table 1** indicates almost 38.5% of PPLELC death attributed to causes other than lung cancer. Therefore, CSS would have more advantages in predicting the prognosis of PPLELC than overall survival. In this study, CSS was the main outcome used to evaluate the prognosis of the SEER and JSPH cohorts. Our study suggested a 5-year CSS rate of 81.8% for stage I–II cases and 56.2% for stage III–IV patients. In contrast, the 5-year survival rates of lung adenocarcinoma were 14.0% and 3.7% for stage III and IV cases, respectively (14). In addition, the SEER and JSPH datasets presented that the TNM stage served as a significant independent prognostic factor for CSS of PPLELC in this study. Consistently, a previous meta-analysis revealed that early-stage diagnosis was a favorable prognosis factor for both overall survival and DFS. Moreover, their systematic review concluded that a better outcome of PPLELC was detected in men and patients who underwent radiotherapy (15).

For NSCLC patients in the early stages, surgery is the only curative treatment. This study confirmed that patients with PPLELC who received surgery had a better prognosis than those who did not. However, most PPLELC cases have no specific clinical manifestations at diagnosis, resulting in a common diagnosis of advanced tumor stages and a missed opportunity for curative resection. Literature reviews suggested effective chemotherapy and radiotherapy for late-stage disease (15, 16). However, our research revealed that chemotherapy and radiotherapy were not correlated with the CSS of PPLELC. Moreover, previous studies have implicated that advanced PPLELC patients benefited little from targeted therapy (17). Unlike classical NSCLC, rare driver genes that were commonly mutated (e.g., EGFR) were detected in PPLELC according to recent reports, which was consistent with our study (18). Eleven tumor samples were tested for gene mutations by NSG, and only three of them carried mutated genes: one had a CYP2D6*10 homozygous mutation T/T and a UGT1A1*6 heterozygous mutation G/A, one had a PAK3 mutation, and one had a TP53 mutation (**Supplementary Table 2**). Ying et al. reported that TP53 (43%) and CYLD (43%) were the two most commonly mutated genes in PPLELC (18), while the frequency of the TP53 mutation rate was revealed to be 6.5% in another cohort (19). The discrepancy may be attributed to the diverse detection methods. Nevertheless, a combination of anti-angiogenic therapy with chemotherapy was reported to be superior to chemotherapy alone in PPLELC of advanced stages (20). According to Bao et al., the median progression-free survival (PFS) was 11.2 months in the former group and only 7.0 months in the latter group (*p* = 0.008). Consistently, the 1-year PFS rates were 41.9% and 17.6%, respectively (20).

The therapeutic efficiency of immune therapy had also been recently reported in several studies. According to a meta-analysis by Tang et al., which included 13 retrospective studies of 1,294 PPLELC cases, positive PD-L1 was observed in 63%–76% of cases. Interestingly, high PD-L1 was more frequent in younger patients (*p* = 0.01) and was correlated with poor DFS (*p* = 0.02) (15). A subsequent study indicated the median PD-L1 expression as 40% in a retrospective cohort from a single medical center (n = 5) (21). Consistently, Fan et al. reported 42.9% (3/7) of PPLELC cases exhibited high PD-L1 expression (18), highlighting that PD-L1 expression was observed in a large proportion of PPLELC cases. Of note, a high proportion of PD-L1 in PPLELC implied immunotherapy as a potential direction in disease treatment. For example, in Wu et al.’s cohort, five PPLELC patients at advanced stages who failed with surgery and chemo-radiotherapy were subjected to immune checkpoint blockade therapy. Three of the patients (3/5, 60%) responded favorably, with the best overall response being partial remission (21). Another case report from Thailand described a patient who received pembrolizumab, a PD-1 inhibitor, with the best response being a stable disease, although the patient died 28 months after diagnosis (22). In the JSPH cohort, one PPLELC patient received chemotherapy combined with immunotherapy and had a stable disease response for 21 months at the end of the follow-up. Nevertheless, prospective clinical studies are necessary to obtain more evidence for PPLELC immunological treatment.

In conclusion, our study described the clinicopathological features, current treatment, and prognosis of PPLELC patients in both the SEER dataset and our medical center. PPLELC is a rare subtype of NSCLC with a higher incidence among Asians and a favorable prognosis. Consistent with other NSCLC subtypes, the TNM stage serves as an independent prognostic factor of PPLELC.

## Data availability statement

The raw data supporting the conclusions of this article will be made available by the authors, without undue reservation.

## Ethics statement

The studies involving human participants were reviewed and approved by Institutional Review Board of The Ethic Committee of Nanjing Medical University First Affiliated Hospital. The patients/participants provided their written informed consent to participate in this study.

## Author contributions

QZ and YD contributed to data curation and writing preparation. LJ, SS, and CL contributed to methodology and formal analysis. RR, WS, and SD contributed to software application and resources provision. WX and HK contributed to project administration and supervision. All authors contributed to the article and approved the submitted version.
