# Supplementary material for: Clinicopathological characteristics and cancer-specific prognosis of primary pulmonary lymphoepithelioma-like carcinoma: a population study of the US SEER database and a Chinese hospital
Source: Front Oncol. 2023 May 19;13:1103169. doi: 10.3389/fonc.2023.1103169 (PMC10235615; doi:10.3389/fonc.2023.1103169)
Supplement: Supplementary file 1 [file Table_1.docx]

Supplemental table 1: Causes of death in PPLELC patients

| Number of death | Percentage (%) | Causes of death |
| --- | --- | --- |
| 24 | 61.5% | Lung cancer |
| 4 | 10.3% | Diseases of Heart |
| 3 | 7.7% | Chronic Obstructive Pulmonary Disease |
| 2 | 5.1% | Symptoms, Signs and Ill-Defined Conditions |
| 1 | 2.6% | Suicide and Self-Inflicted Injury |
| 1 | 2.6% | Cerebrovascular Diseases |
| 1 | 2.6% | Oropharynx |
| 1 | 2.6% | Melanoma of the Skin |
| 1 | 2.6% | In situ, benign or unknown behavior neoplasm |
| 1 | 2.6% | Other Cause of Death |

Supplemental table 2: The mutation information of 11 PPLELC patients

| Patient ID | Age | Sex | Cohort | Gene mutation |
| --- | --- | --- | --- | --- |
| 1 | 48 | Male | JSPH | No mutation |
| 2 | 65 | Male | JSPH | CYP2D6*10 (T/T), UGT1A1*6 (G/A) |
| 3 | 66 | Female | JSPH | No mutation |
| 4 | 59 | Female | JSPH | No mutation |
| 5 | 61 | Male | JSPH | No mutation |
| 6 | 55 | Female | JSPH | No mutation |
| 7 | 50 | Female | JSPH | PAK3 P.L233fs |
| 8 | 64 | Male | JSPH | TP53 P.R273H |
| 9 | 46 | Female | JSPH | No mutation |
| 10 | 74 | Female | JSPH | No mutation |
| 11 | 55 | Female | JSPH | No mutation |
